# Phosphorylation of the amino-terminus of the AGC kinase Gad8 prevents its interaction with TORC2

**DOI:** 10.1098/rsob.150189

**Published:** 2016-03-02

**Authors:** Wei Du, Gabriella M. Forte, Duncan Smith, Janni Petersen

**Affiliations:** 1 Faculty of Life Sciences, University of Manchester, Oxford Road, Manchester M13 9PT, UK; 2 Flinders Centre for Innovation in Cancer, School of Medicine, Flinders University, Adelaide, South Australia 5001, Australia; 3 South Australia Health and Medical Research Institute, North Terrace, PO Box 11060, Adelaide, South Australia 5000, Australia; 4 Biological Mass Spectrometry, Cancer Research UK Manchester Institute, The Paterson Building, Wilmslow Road, Manchester M20 4BX, UK

**Keywords:** TORC2, Gad8, PKC, SGK1, *Schizosaccharomyces pombe*

## Abstract

Cell proliferation, metabolism, migration and survival are coordinated through the tight control of two target of rapamycin (TOR) kinase complexes: TORC1 and TORC2. Here, we show that a novel phosphorylation of fission yeast Gad8 (AGC kinase) on the evolutionarily conserved threonine 6 (Thr6) prevents the physical association between Gad8 and TORC2. Accordingly, this block to protein interactions by Gad8 Thr6 phosphorylation decreases TORC2-controlled activation of Gad8. Likewise, phosphorylation of Gad8 Thr6, possibly by PKC, prevents the association of Gad8 with TORC2 thereby increasing TORC2 activity, because it reduces Gad8-mediated feedback inhibition of TORC2. Consistently, the introduction of a Gad8 T6D mutant, that mimics phosphorylation, increased TORC2 activity. Increased PKC^Pck2^ expression prevented Gad8–TORC2 binding and so reduced the TORC2-mediated phosphorylation of Gad8 serine 546 that activates Gad8. Interestingly, independent of the Ser546 phosphorylation status, Gad8 Thr6 phosphorylation is important for remodelling the actin cytoskeleton and survival upon potassium ion and heat stresses. In contrast, Ser546 phosphorylation is required for the control of G1 arrest, mating, cell length at division and vascular size. Finally, these findings reveal a novel mode of TORC2 activation that is essential for cell survival following stress.

## 1. Introduction

Target of rapamycin (TOR) is a highly conserved protein kinase that coordinates cell growth and proliferation with changes in the cellular environment and stress [1]. In all organisms, TOR forms two structurally and functionally distinct multi-protein complexes, TORC1 and TORC2. These complexes are defined by unique interacting components that are highly conserved across species; in mammalian cells, regulatory-associated protein of mTOR (Raptor) defines TORC1, whereas the TORC2 complex uniquely incorporates rapamycin-insensitive companion of mTOR (Rictor) and Sin1 [2–4]. Mip1 and Ste20 represent the fission yeast homologues of Raptor and Rictor, respectively [5]. The unique TORC1 and TORC2 interacting proteins are thought to provide substrate specificity for each individual TOR complex [6].

Fission yeast contains two TOR kinases; Tor2 is the main TORC1 kinase, whereas the majority of TORC2 incorporates the non-essential Tor1 kinase [7–9]. In general, because TORC1 is sensitive to rapamycin [10], this has accelerated the understanding of the TORC1 signalling. TORC1 is essential for anabolic growth and proliferation [11]. In contrast, TORC2 regulation and signalling are less well understood. TORC2 has been shown to control metabolism, the cytoskeleton and cell survival after exposure to stress [10].

The fission yeast Gad8 protein is a member of the AGC subfamily of protein kinases (reviewed in [12]) and is regulated by TORC2. Phosphorylation of the T-loop of Gad8 by PDK1^Ksg1^ activates the kinase; however, full Gad8 activation requires additional TORC2-dependent phosphorylation within its carboxy-terminus [13,14]. Environmentally induced enhancement of PDK1^Ksg1^ activity boosts Gad8 activity [14] to promote a conserved feedback loop that downregulates TORC2 activity, because phosphorylation of the TORC2 ATP binding site by Gad8 reduces TORC2 activity [15]. Importantly, simultaneous Gad8 activation by TORC2 and Gad8 feedback inhibition of TORC2 would constitute a futile cycle as the two modifications negate one another. It is therefore highly likely that additional mechanisms provide tighter regulation of this negative feedback loop. We have previously shown that Gad8 ser546 phosphorylation is very dynamic as it is dephosphorylated within minutes of TORC2 inhibition. In contrast, dephosphorylation of the TORC2 ATP binding site, to increase TORC2 activity, occurs with slower kinetics [15].

We now show that phosphorylation of fission yeast Gad8 on the conserved residue Thr6 reduces the physical interaction between Gad8 and TORC2 to decrease both Gad8-mediated inhibition of TORC2 along with TORC2-controlled activation of Gad8. Thr6 represents a novel PKC consensus phosphorylation site that is conserved in a subset of AGC kinases, including mammalian SGK1/2 and the *Saccharomyces cerevisiae* kinases Ypk1 and Sch9. Our findings reveal a novel mode of TORC2 activation that is important for actin remodelling and survival following either potassium ion or heat stress. Finally, we show that Gad8 Ser546 phosphorylation is required for the control of G1 arrest, mating, cell length at division and vascular size, but not for remodelling of the actin cytoskeleton and survival upon potassium ion and heat stresses.

## 2. Results

### 2.1. The evolutionarily conserved Thr6 of Gad8 is phosphorylated

**Figure 1.**
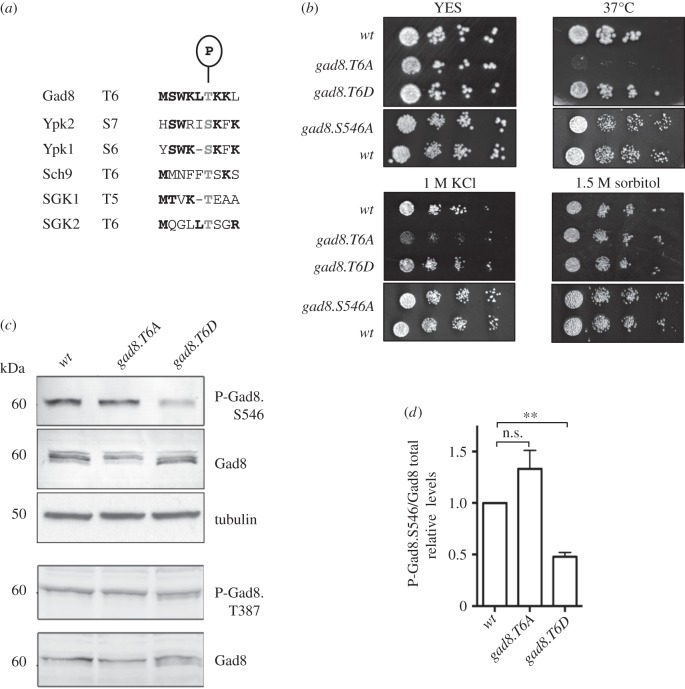
Gad8 Thr6 is phosphorylated. (*a*) Gad8 Thr6 is evolutionarily conserved. (*b*) Stress response of *gad8* mutants. (*c*) Gad8 protein and Thr387 and Ser546 phosphorylation levels in *T6A* and *T6D* mutants. (*d*) Relative Gad8 Ser546 phosphorylation levels in *T6A* and *T6D* mutants.

We have previously shown that environmentally induced enhancement of PDK1^Ksg1^ activity boosts Gad8 activity to promote a conserved feedback loop that downregulates TORC2 activity, because phosphorylation of the TORC2 ATP binding site by Gad8 reduces kinase activity [15]. In addition, we found that Gad8 is heavily phosphorylated [15]. To gain further insights into the functional consequences of Gad8 phosphorylation, and its role in Gad8 control of TORC2 activity, Gad8 was immunoprecipitated from wild-type fission yeast cells, and sites of Gad8 phosphorylation were identified via tandem mass spectrometry. Two well-characterized sites of phosphorylation were identified, the PDK1^Ksg1^ controlled T-loop residue Thr387 and the TORC2-controlled Ser546 [14] (data not shown) alongside a novel phosphorylation event at threonine 6 (figure 1*a* and electronic supplementary material, figure S1A). Thr6 is conserved in the homologous kinases in *S. cerevisiae* (Ypk1p, Ypk2p and Sch9p) and humans (SGK1 and SGK2; figure 1*a*), highlighting the potential for a novel, conserved, mode of regulation that exploits phosphorylation of Thr6 within this family of AGC kinases. Gad8 is also an AKT homologue; however, the amino-terminal Thr6 is not conserved in AKT.

### 2.2. Gad8 Thr6 regulates cell survival following heat and potassium ion stress

The TORC2 Gad8 signalling pathway is required for tolerance of a variety of stresses [14,16–18]. To assess the significance of phosphorylation on *Schizosaccharomyces pombe* Gad8 Thr6 in the response to environmental stress, it was mutated to either alanine (T6A), to block signalling, or aspartic acid (T6D), to mimic constitutive phosphorylation. Interestingly, the *gad8.T6A* mutant was specifically sensitive to growth at increased temperature and was slow growing upon exposure to KCl (figure 1*b*). Neither *gad8.T6A* nor *gad8.T6D* altered tolerance to other stresses including: hydrogen peroxide, CaCl_2_ and sorbitol-induced osmotic stress (electronic supplementary material, figure S1B). Because Gad8 activity is required for cell growth in response to all these stresses [14,16], our initial data suggested that Thr6 phosphorylation moderately attenuated Gad8 function, rather than completely blocking its kinase activity.

### 2.3. Gad8 Thr6 phosphorylation affects Gad8 S546 phosphorylation

We next assessed the impact of Gad8 Thr6 phosphorylation on Gad8 protein levels and phosphorylation. Gad8 protein levels were slightly reduced in *gad8.T6A* mutant cells (figure 1*c* and electronic supplementary material, figure S1C,D); however, Gad8 protein stability and turnover were unaffected by the phosphorylation status of Thr6 (electronic supplementary material, figure 1SE,F). As mentioned previously, PDK1^Ksg1^ phosphorylation of the T-loop of Gad8, at position Thr387, is essential for kinase activity, whereas additional TORC2-dependent phosphorylation at the carboxy-terminal further enhances kinase activity. The TORC2-controlled phosphorylation of Gad8 Ser546 is equivalent to the mTORC2-controlled phosphorylation of AKT on the hydrophobic motif (Ser473), which activates AKT in mammals [13,19]. The relative level of Ser546 phosphorylation was reduced in the *gad8.T6D* mutant (figure 1*c*,*d*). In contrast, the PDK1^Ksg1^-dependent phosphorylation at Thr387 was unaffected by Thr6 phosphorylation status (figure 1*c*), suggesting that the impact of Thr6 phosphorylation on additional Gad8 phosphorylation events is specific to the TORC2-dependent regulation of Gad8.

TORC2 deletion mutants (*tor1.Δ, ste20.Δ* and *sin1.Δ*) are all sensitive to increased temperature and potassium levels [14,16,17]. As mentioned above, the sensitivity to either heat or potassium stress was unaffected by the *gad8.T6D* mutation (figure 1*b*), yet TORC2-controlled Ser546 phosphorylation is reduced in the *gad8.T6D* mutant (figure 1*c*,*d*). Exposing the *gad8.S546A* mutant to heat and potassium stress established that Gad8 Ser546 phosphorylation is not critical for cell survival following heat or potassium stress, because the *gad8.S546A* mutant showed no growth defect compared with wild-type cells when exposed to these stresses (figure 1*b* and table 1). In contrast, *gad8.T6A* mutant cells with normal levels of Ser546 phosphorylation were sensitive to heat and potassium stress (figure 1*b* and table 1). The mechanistic basis for enhanced sensitivity to heat or potassium stress of the *gad8.T6A* mutant is currently unclear.

**Table 1.** Summary of the effect of Gad8 Thr6, S546 and kinase dead mutants on Gad8 function.

phenotype	*gad8.T6A*	*gad8.T6D*	*gad8.S546A*	gad8.KD*
Gad8.S546 phosphorylation	unaffected	reduced	n.a.	increased (*KD*: kinase dead mutant)
*Tor1 activity*	unaffected	increased	increased	increased
the T6 phenotype is S546-dependent and similar to Gad8.KD (Gad8 activity is likely to determine phenotype)				
cell length at division	reduced	increased	increased	increased
mating efficiency	unaffected	reduced	reduced	reduced
G1 arrest	delayed	small delay	delayed	delayed
the T6 phenotype is S546-dependent but opposite to Gad8.KD. (TORC2 activity is likely to determine phenotype, but sensitive to lack of Gad8 activity)				
vacuolar size	reduced	increased	increased	reduced
the T6 phenotype is S546 independent but similar to Gad8.KD (T6-dependent phenotype but sensitive to lack of Gad8 and increased TORC2 activities)				
NETO	unaffected	reduced	unaffected	reduced
37° stress	sensitive	unaffected	unaffected	sensitive
KCl stress	slow growth	unaffected	unaffected	sensitive
phenotype is T6 independent				
osmotic stress	unaffected	unaffected	unaffected	sensitive
oxidative stress	unaffected	unaffected	unaffected	sensitive
CaCl_2_ stress	unaffected	unaffected	unaffected	sensitive
nutrient stress	unaffected	unaffected	slight sensitivity	sensitive

### 2.4. Thr6 affects cell and vacuolar size through its impact upon Ser546 phosphorylation

**Figure 2.**
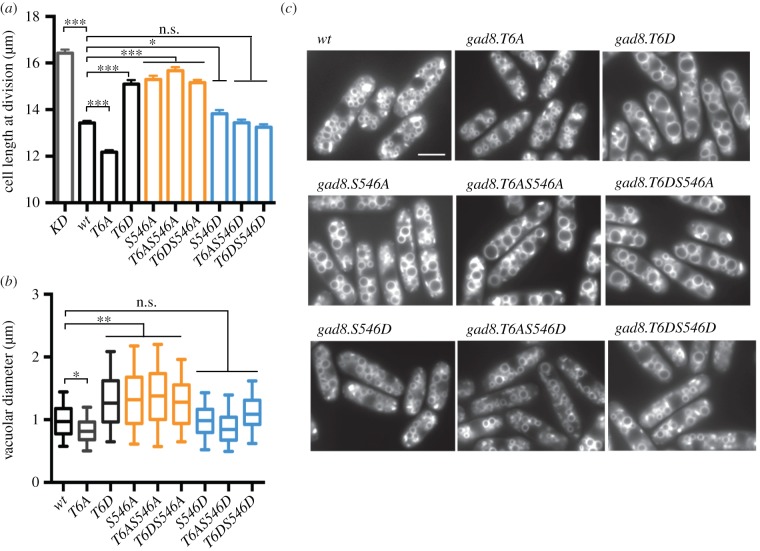
Gad8 Thr6 regulates cell and vacuolar size through its effect on Ser546 phosphorylation. (*a*) Cell length at division of indicated *gad8* mutants. (*b*) Vacuolar size of indicated *gad8* mutants. (*c*) FM4-64 staining of vacuoles in indicated *gad8* mutants.

Cell size at division is increased in *gad8* deficient mutants. Cell size at division is also increased in the TORC2 deficient deletion mutants *tor1.Δ, ste20.Δ* and *sin1.Δ*, indicating that the G2/M transition is delayed when either Gad8 or TORC2 function is compromised [16,18]. Although the molecular mechanisms are currently unclear, the delay to mitotic commitment that underlies the increase in size at division of *gad8* deficient mutants suggests that Gad8 plays a key role in regulating the timing of the G2/M transition [16,18]. To assess the impact of Thr6 phosphorylation upon cell size control, we measured cell size at division of Thr6 mutants. The cell size at division of *gad8.T6D* mimicked the kinase dead *gad8.KD* and the TORC2 deficient deletion mutants *tor1.Δ, ste20.Δ* and *sin1.Δ* in being increased (figure 2*a* and table 1) [16,18]*.* Conversely, cell size at division was reduced in *gad8.T6A* (figure 2*a*). The *gad8.S546A* mutation phenocopied the increased cell length at division of *gad8.T6D* (figure 2*a* and table 1). Therefore, the increase in size at division of *gad8.T6D* mutants may stem from their reduction in Ser546 phosphorylation (figure 1*c*). To test this possibility, the cell size of different combinations of Thr6 and Ser546 double mutants were measured (figure 2*a*). Importantly, the *gad8.T6A-S546A* double mutant resembled *gad8.S546A,* as both appeared elongated. Together, these data suggest that the cell size at division of Gad8 Thr6 mutants is indeed dictated by the status of TORC2-mediated phosphorylation of serine 546 on Gad8.

The nutritional environment also determines cell size at division of fission yeast [20,21]. Gad8 is essential for the advancement of mitosis that leads to cell division at a reduced cell length following nutrient stress [18]. However, Thr6 phosphorylation does not appear to be required for this nutrient stress-induced advancement of mitosis and cell division (electronic supplementary material, figure S2*a*).

The TORC2 mutants *tor1, ste20, sin1* and *gad8* mutants are all required for vacuolar (yeast lysosomes) integrity and size [19]. Consequently, the vacuoles of cells lacking *gad8*.*Δ* are small and fragmented [19]. We therefore assessed the impact of Thr6 phosphorylation status upon vacuolar size. Vacuoles were enlarged in *gad8.T6D* (figure 2*b*,*c* and table 1) and shrunken in *gad8.T6A* mutants. The *gad8.S546A* mutant also induced an increase in vacuole size. Examination of double mutants, that combined mutations at Ser546 with Thr6 mutants, established that vacuolar size is also determined by the phosphorylation status of Ser546 (figure 2*b*,*c*).

The ability to form autophagosomes and so survive nitrogen or glucose starvation might be influenced by altered vacuolar function. However, all *gad8* Thr6 mutants survived nitrogen starvation. Autophagy in fission yeast is therefore unlikely to be affected by Thr6 phosphorylation status (electronic supplementary material, figure S2*b*). In summary, the Thr6 phosphorylation-dependent impact on cell length and vacuole size appears to be mediated via TORC2-controlled Ser546 phosphorylation of Gad8.

### 2.5. Gad8 Thr6 phosphorylation is important for sexual differentiation

**Figure 3.**
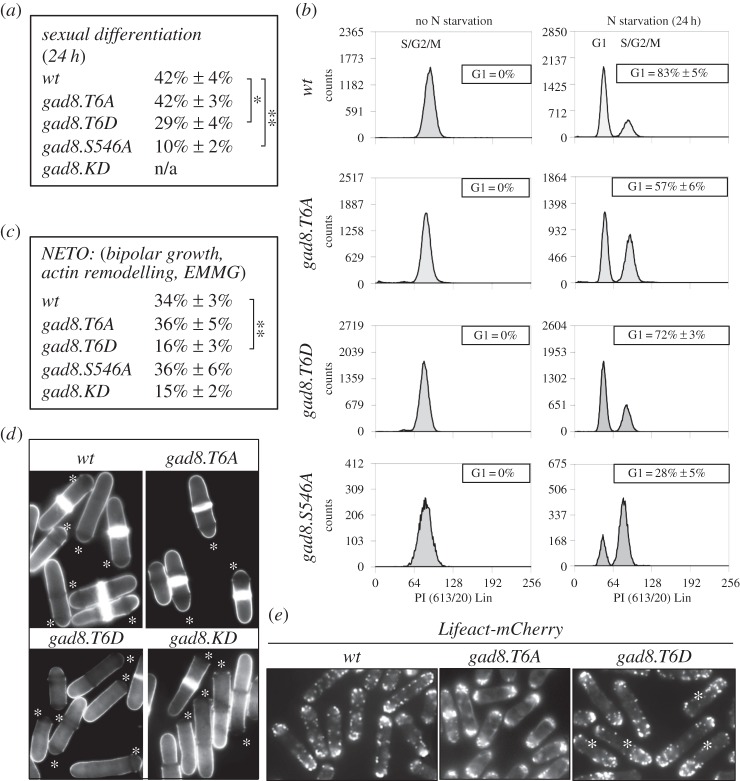
Gad8 Thr6 regulates sexual differentiation and F-actin re-modelling. (*a*) Sexual differentiation efficiency assay. (*b*) The efficiency of G1 arrest in *gad8* mutants when starved for nitrogen. The data shown are from a single representative experiment out of three repeats. (*c*) The frequency of NETO of indicated *gad8* mutants. (*d*) Calcofluor staining of indicated *gad8* mutants, the asterisk marks the new cell ends. (*e*) *Lifeact-mCherry* is used to visualize F-actin in the *gad8 thr6 Lifeact-mCherry* double mutants, the asterisk marks long mono-polar cells in the *gad8.T6D* mutants.

Gad8 was initially identified in fission yeast as a *G*1 *a*rrest *d*eficient (gad) mutant. Consequently, *gad8.KD* mutants are unable to undergo sexual differentiation and mating and are therefore sterile [14]. Flow cytometric analysis of DNA content in wild-type fission yeast cultures, grown in a good nitrogen source, does not reveal a G1 population [22]. However, 24 h of nitrogen starvation induces G1 arrest in 80% of wild-type cells and, when mixed with cells of the opposite mating type, 40% of wild-type cells undergo sexual differentiation and mating (figure 3*a*,*b*). Both G1 arrest and sexual differentiation were reduced by 75% in the *gad8.S546A* mutant (figure 3*a*,*b*). In the *gad8.T6A* mutant, the level of G1 arrests was reduced by 25% (figure 3*b*). Nevertheless, the mating efficiency of *gad8.T6A* mutants was similar to wild-type cells. Therefore, once arrested in G1, *gad8.T6A* mutants mate efficiently (figure 3*a*). In contrast, sexual differentiation and mating were reduced in *gad8.T6D* mutant even though 70% of cells arrested in G1 (figure 3*a*,*b*). Thus, Gad8 regulates G1 arrest and sexual differentiation via independent mechanisms yet regulation through phosphorylation of Thr6 is important for both functions (table 1).

### 2.6. Gad8 Thr6 phosphorylation is important for remodelling of the F-actin cytoskeleton

Following the induction of sexual differentiation, the cell must change the positioning of the growth zone (projection tip) to initiate the growth at new sites that will support growth towards a mating partner. This switch in actin organization is determined by a signalling cascade that is triggered by a gradient of sex pheromones [23–26]. Consequently, the actin cytoskeleton is continuously reorganized during courtship before being set in a new polarized pattern typical of conjugating cells [25]. It has already been established that *S. cerevisiae* Ypk1p and human SGK1 regulate actin organization [27,28]. Because the reduction in the mating efficiency of the *gad8.T6D* mutant cannot be explained by a defect in the competence to arrest cell cycle progression in G1 (figure 3*b*), we explored the possibility that it may arise from inefficient reorganization of the F-actin cytoskeleton by monitoring F-actin remodelling in Thr6 mutants.

Fission yeast cells are rod shaped and grow by tip extension. A ‘newborn’ fission yeast cell grows in a mono-polar fashion from the old cell end. Mid-way through G2, the actin cytoskeleton and the growth machinery is activated at the new cell end (new end take off, NETO) to initiate a phase of bipolar growth that persists until division [29]. Therefore, NETO can be used as a proxy for the ability to reorganize actin in fission yeast. Calcofluor can be used to visualize NETO, as the growing ends of fission yeast cells stain more intensely with this fluorescent dye. The number of cells undergoing NETO (bright at both cell ends) in the *gad8.KD* kinase dead and *gad8.T6D* mutants was reduced, whereas both *gad8.T6A* and *gad8.S546A* cells were NETO proficient (figure 3*c* and table; figure 3*d*, asterisks indicate the new cell ends). We next used *Lifeact-mCherry* to visualize F-actin [30]. The presence of long cells in the *gad8.T6D Lifeact-mCherry* double mutant, which had F-actin localized at the old end only, confirmed that *gad8.T6D* cells are NETO deficient (figure 3*e*). In summary, the reduced mating efficiency of *gad8.T6D* cells correlated with a reduction in the ability to reorganize the F-actin cytoskeleton.

### 2.7. Gad8 Thr6 phosphorylation specifically reduces TORC2-controlled activation of Gad8 and Gad8-mediated feedback inhibition of TORC2

Our phenotypic analyses suggest that Gad8 Thr6 phosphorylation is required for the execution of only a subset of Gad8 functions. Gad8 Thr6 phosphorylation is important for cell survival upon heat and potassium stresses and F-actin reorganization in mating. For the reorganization of the F-actin cytoskeleton, this requirement is independent of the Ser546 phosphorylation status. To gain further insights into the mechanistic basis for this specificity of Thr6 on Gad8 function, we assessed Gad8 activity and the ability of the Thr6 mutant cells to regulate known Gad8 substrates. We began by developing an *in vitro* assay to monitor Gad8 kinase activity. We have previously reported direct phosphorylation of Tor1.T1972 as part of TORC2 by Gad8 [15]. Thus, a small fragment of Tor1 fused to GST was expressed in, and isolated from, *Escherichia coli* for use as *in vitro* substrate. Phospho-specific Tor1.T1972 antibodies measured Gad8 activity towards T1972 on this recombinant Tor1 substrate. A twofold serial dilution of immunoprecipitated wild-type Gad8 established that our *in vitro* assays monitored kinase activity within a linear range (electronic supplementary material, figure S3A). Thr6 phosphorylation status did not affect Gad8 *in vitro* kinase activity towards the Tor1–GST fusion protein (electronic supplementary material, figure S3B).

**Figure 4.**
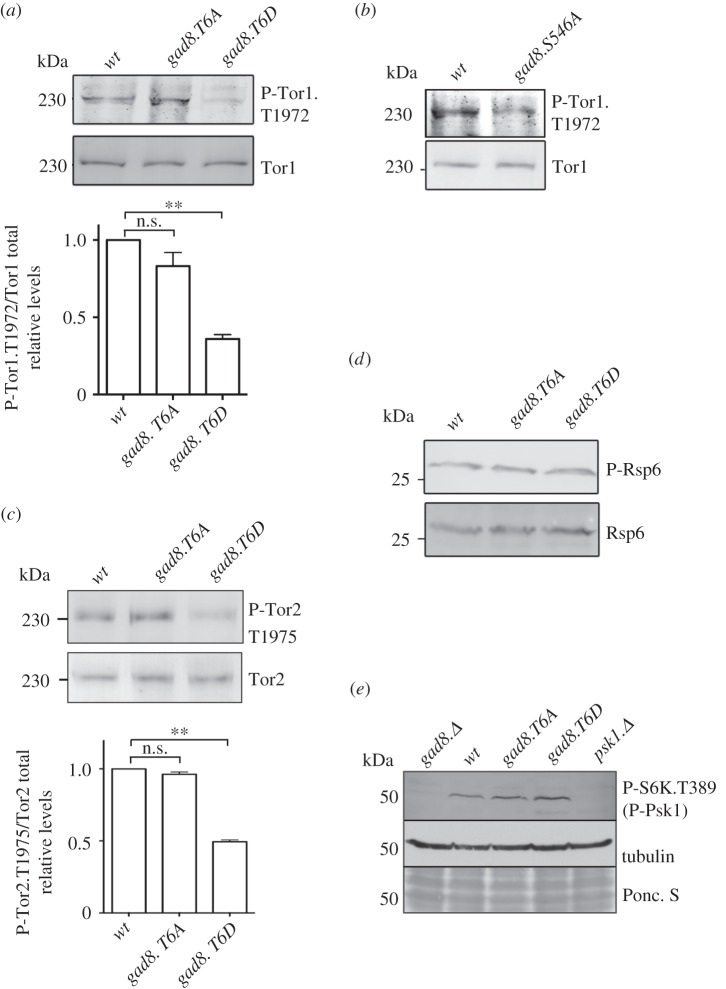
Gad8 Thr6 phosphorylation reduces Gad8-controlled phosphorylation of Tor1 and Tor2. (*a*) Gad8 phosphorylation of the Gad8-specific substrate Tor1.T1972 in *T6A* and *T6D* mutants, along with quantification of relative Tor1.T1972 phosphorylation levels. (*b*) Gad8 phosphorylation of the Gad8-specific substrate Tor1.T1972 in the *S546A* mutant. (*c*) Gad8 phosphorylation of the Gad8-specific substrate Tor2.T1975 in *T6A* and *T6D* mutants, along with quantification of relative Tor2.T1975 phosphorylation levels. (*d*) Gad8-dependent phosphorylation of Rsp6 in *T6A* and *T6D* mutants. (*e*) Gad8 phosphorylation of the Gad8-dependent substrate Psk1 in *T6A* and *T6D* mutants.

We next assessed Gad8 *in vivo* kinase activity. The Gad8-dependent *in vivo* phosphorylation of Tor1.T1972 was reduced in the *gad8.T6D* and *gad8.S546A* mutants (figure 4*a*,*b*). In contrast, the Tor1.T1972 phosphorylation status was not altered by the *gad8.T6A* mutation (figure 4*a*). Fission yeast has two TOR kinases [31]; the Gad8-dependent phosphorylation of Tor1 Thr1972 is conserved for Tor2 (the main kinase of TORC1) [7–9] as phosphorylation of Tor2 Thr1975 also is Gad8 dependent [15]. Tor2.T1975 phosphorylation resembled Tor1.T1972 phosphorylation in also being reduced by the *gad8.T6D* mutation (figure 4*c*). Interestingly, the Thr6 mutants had no impact on two additional Gad8-dependent phosphorylation events *in vivo* (figure 4*d*,*e*). The *S. pombe* ribosome protein 6 (Rps6) is a homologue of the S6 protein that is phosphorylated by mammalian S6 kinase [32]. We have previously shown that phosphorylation of Rps6 is regulated by Gad8 [33]. Mutation of Thr6 had no impact upon Gad8-controlled Rsp6 phosphorylation (figure 4*d*). In fission yeast, Rsp6 phosphorylation is also regulated by TORC1 and the Psk1 kinase; furthermore, the activation of Psk1 is TORC1 dependent [34]. Interestingly, we found that this TORC1-dependent phosphorylation of Psk1 also required Gad8 (figure 4*e*). This dependency for Gad8 in regulation of Psk1 activation may provide an explanation for the role of Gad8 and TORC2 in S6 phosphorylation. However, importantly, the Gad8-dependent Psk1 phosphorylation was unaffected by the phosphorylation status of Thr6.

Together, our data suggest that Thr6 phosphorylation has no impact on *in vitro* kinase activity and no impact on two *in vivo* phosphorylation sites (on Rsp6 and Psk1) regulated by Gad8. However, Thr6 affects the reciprocal regulation of TORC2 and Gad8 activities *in vivo*, because both Gad8.S546 (figure 1*c*) and Tor1.T1972 (figure 4*a*) phosphorylation are reduced in *gad8.T6D* mutants.

### 2.8. Thr6 phosphorylation prevents physical association between Gad8 and TORC2

**Figure 5.**
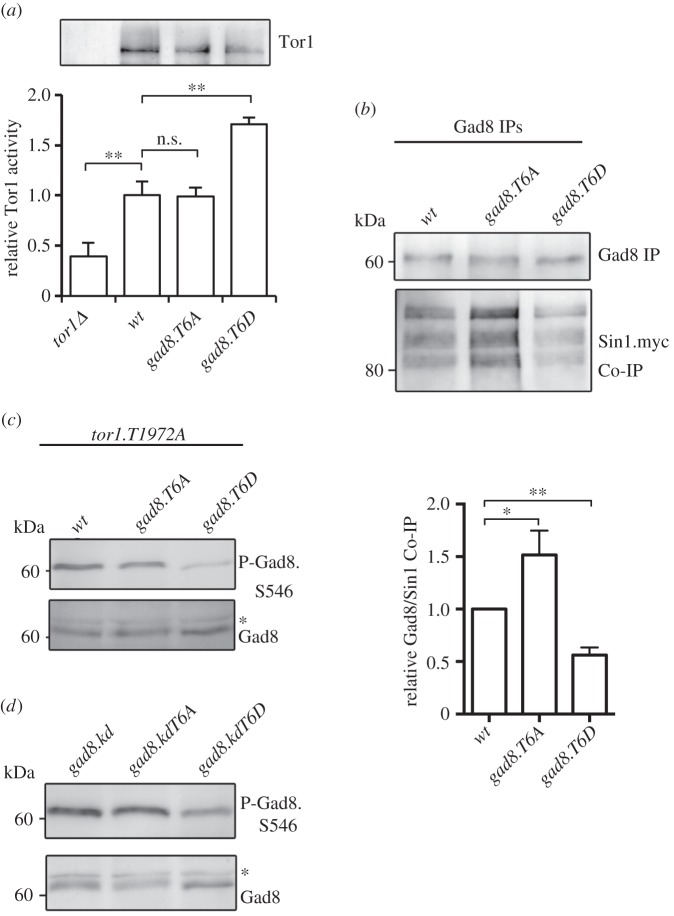
Gad8 Thr6 phosphorylation reduces TORC2 and Gad8 interactions. (*a*) Tor1 *in vitro* kinase assays. Immunoprecipitated Tor1 from the *gad8.T6D* mutant has a higher Tor1 kinase activity than wild-type Gad8. (*b*) Gad8 was immunoprecipitated from *T6A* and *T6D* mutant and Sin1 (TORC2) co-immunoprecipitation measured. (*c*) TORC2 phosphorylation of the Gad8 Ser546 in hyperactive *tor1.T1972A and T6A* and *T6D* double mutants. (*d*) TORC2 phosphorylation of the Gad8 Ser546 in *gad8.KD* kinase dead *and T6A* and *T6D* double mutants. (*b,c*) Asterisk indicates a background band.

Because phosphorylation of Tor1.T1972 by Gad8 is inhibitory for TORC2 functions [15], the reduction in Tor1.T1972 phosphorylation in the *gad8.T6D* mutant (figure 4*a*) is likely to elevate TORC2 activity. To test this possibility, we used established assays to measure *in vitro* Tor1 kinase activity in the Thr6 mutants [15,35]. As anticipated by the reduction in T1972 phosphorylation, *in vitro* Tor1 activity increased in the *gad8.T6D* mutant (figure 5*a*), yet was unaffected in the *gad8.T6A* mutant. This *gad8.T6D-*dependent increase in *in vitro* TORC2 activity is in stark contrast to the observed reduction in TORC2-dependent Gad8.S546 phosphorylation (figure 1*c*).

To gain more insight into this conundrum, we next considered the possibility that Thr6 phosphorylation may decrease the reciprocal Gad8–TORC2 regulation by compromising the physical interaction between Gad8 and TORC2. We therefore assessed the interaction between Gad8 and the TORC2-specific component Sin1 [5]. An increase in the levels of TORC2-Sin1 co-precipitating with Gad8.T6A mutant protein, and a converse reduction in the association in a Gad8.T6D precipitation, suggested that Thr6 phosphorylation status does indeed regulate the interaction between Gad8 and TORC2-Sin1 (figure 5*b*).

To further validate the role of Thr6 in regulating Gad8's interaction with TORC2, we generated a *tor1.T1972A gad8.T6D* double mutant. We have previously established that *tor1.T1972A* mutants have increased TORC2 activity, because Tor1.T1972A cannot be inhibited by Gad8. Consequently, *tor1.T1972A* mutants have increased Gad8.S546 phosphorylation [15]. Consistent with the reduced binding of *gad8.T6D* to TORC2-Sin1 (figure 5*b*), Gad8.S546 phosphorylation was still reduced in the *tor1.T1972A gad8.T6D* double mutant (figure 5*c*) despite the increase in kinase activity of Tor1.T1972A mutant proteins. We next took advantage of the catalytically inactivating *gad8.KD kinase dead* mutant, in which TORC2 activity also is increased, because the loss of Gad8 activity blocks its ability to inhibit TORC2 [15]. Introducing this kinase dead mutation alongside the T6D in a *gad8.KD-T6D* double mutant also failed to rescue the reduced Gad8.S546 phosphorylation of *gad8.T6D* mutants (figure 5*d*). Together, these data suggest that elevated TORC2 activity cannot rescue the lower levels of Ser546 phosphorylation in the *gad8.T6D* mutant.

In summary, the *gad8.T6D* mutation reduced the association between Gad8 and TORC2-Sin1. This reduced protein interaction can explain the specific inability of *gad8.T6D* to regulate T1972 phosphorylation of Tor1 (TORC2) (figure 4*a*) and the inability of TORC2 to regulate Gad8.S546 in the *gad8.T6D* mutant (figure 1*c*).

### 2.9. Thr6 lies within a protein kinase C consensus site

**Figure 6.**
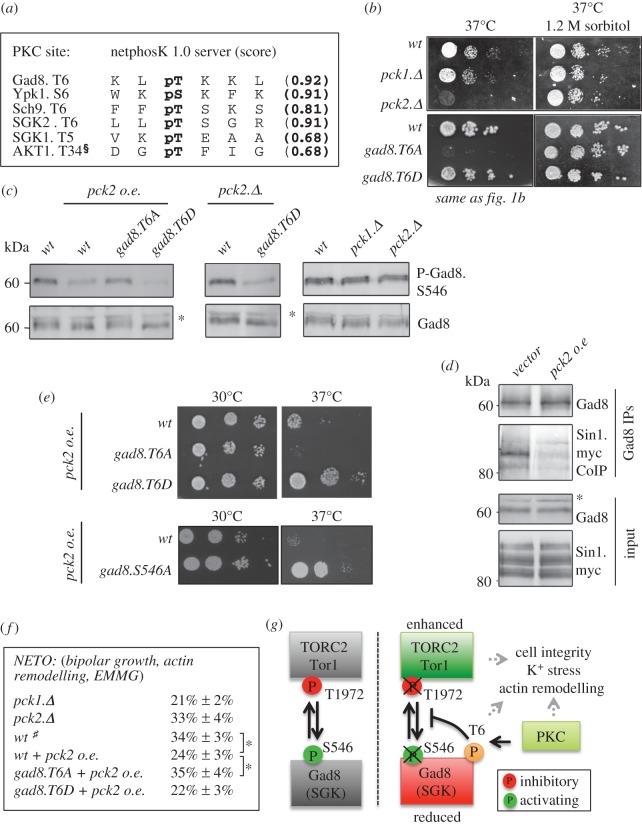
High levels of PKC reduce Gad8 binding to TORC2-Sin1. (*a*) Gad8.Thr6 resembles a protein kinase C phosphorylation motif that is conserved in other AGC kinases. §Has previously been identified by Powell *et al.* [37]. (*b*) Heat stress sensitivity of *pck2.Δ* and *gad8.T6A* mutants is rescued by osmotic stabilizer. (*c*) Pck2 overexpression in *T6A* and *T6D* mutants. Pck2 reduces Gad8.S546 phosphorylation through Thr6. (*d*) Pck2 overexpression reduces Gad8 and Sin1 (TORC2) co-immunoprecipitation. (*e*) Stress response of *gad8 T6A*, *T6D* and *S546A* mutants following Pck2 overexpression. (*f*) Measurement of actin remodelling (NETO, new end take off) in *T6A* and *T6D* mutants. Hash symbol denotes identical to figure 3*c*. (*g*) Schematic suggesting that PKC-dependent regulation of Gad8 Thr6 blocks Gad8 binding to TORC2 and therefore reduces Gad8-dependent TORC2 inhibition and TORC2 activation of Gad8. (*c*,*d*) Asterisk indicates a background band.

Together, our data suggest that the kinase that phosphorylates Thr6 regulates a subset of TORC2- and Gad8-dependent activities. To identify the candidate Thr6 kinase, we submitted the Gad8 sequence to the NetPhosK 1.0 server [36]. Thr6 of Gad8 along with the sites found in the Gad8 homologues, YPK1, Sch9 and SGK kinases, all conformed to the consensus for phosphorylation by protein kinase C (PKC; figure 6*a*). In mammalian cells, the Gad8 homologue AKT has been shown to be phosphorylated by PKC on Thr34 (figure 6*a*) [37,38]. AKT.T34 is found within the membrane binding PH domain of the kinase (electronic supplementary material, figure S4A). Thr34 phosphorylation prevents the recruitment of AKT to membranes [37]. Importantly, none of the amino terminal PKC consensus sites in Gad8, Ypk1 and Sch9 lie within the respective membrane binding regions (electronic supplementary material, figure S4A). The mammalian homologues, the SGKs, do not have a predicted membrane-binding domain (electronic supplementary material, figure S4A). Interestingly, the Thr6-regulated processes (potassium sensitivity, actin cytoskeleton and heat stress that activates the cell wall integrity pathway) are all regulated by the fission yeast PKC homologues PKC^Pck1^ and PKC^Pck2^ [39,40].

### 2.10. PKC^Pck2^ reduces Gad8 S546 phosphorylation

In fission yeast, several PKC^Pck1^ and PKC^Pck2^ functions are redundant. However, only *pck2.Δ* mutants are sensitive to heat stress (figure 6*b*). The rescue of this heat sensitivity by the addition of sorbitol to buffer against osmotic shock indicates that the temperature sensitivity of *pck2*.Δ deletion cells arises from cell lysis at the elevated temperature (figure 6*b*). Sorbitol rescued the heat stress sensitivity (figures 1*b* and 6*b*) and cell lysis at high temperatures of *gad8.T6A* mutant cells as well (electronic supplementary material, figure S4B). While the *gad8.T6D* mutant was not sensitive to heat stress (figure 1*c*), a *gad8.T6D pck2.Δ* double mutant was (electronic supplementary material, figure S4C), implying that PKC^Pck2^ has multiple targets that are important for cell integrity at high temperatures. One prediction of increased phosphorylation of Gad8 Thr6 by PKC would be a decrease in Gad8 Ser546 phosphorylation, whereas complete loss of PKC would be predicted to have very limited impact, because the *gad8.T6A* mutant has no impact on Gad8 S546 phosphorylation (figure 1*c*). PKC^Pck2^ was ectopically expressed from the *nmt42* promoter [41]. Increased PKC^Pck2^ expression in wild-type cells, but not in the *gad8.T6A* mutant, reduced Ser546 phosphorylation, whereas deletion of either *pck1.Δ* or *pck2.Δ* had no impact on Ser546 phosphorylation levels (figure 6*c*). Importantly, this PKC^Pck2^ expression rescued the temperature sensitivity of *pck2.Δ* mutant (electronic supplementary material, figure S4D). PKC^Pck2^ is known to regulate the Pmk1 MAPK kinase required for cell integrity [42] and Pmk1 has been linked to TORC2 regulation [43]. However, expression of PKC^Pck2^ in a *pmk1.Δ* still reduced Gad8 Ser546 phosphorylation (electronic supplementary material, figure S4E). Together, our data suggest that PKC^Pck2^ influences Gad8 Ser546 phosphorylation status as an indirect consequence of its phosphorylation of Gad8 Thr6.

### 2.11. PKC^Pck2^ prevents Gad8–TORC2 binding

A second prediction arising from PKC^Pck2^-dependent regulation of Thr6 is that elevation of Pck2 levels will reduce Gad8 binding to TORC2-Sin1. Gad8 was therefore immunoprecipitated from cells expressing PKC^Pck2^ ectopically (*pkc2* oe) and the amount of Sin1 co-immunoprecipitating with Gad8 was determined by Western blotting. In a phenocopy of *gad8.T6D* mutant cells, PKC^Pck2^ expression reduced the amount of Sin1 binding to Gad8 (figure 6*d*). Furthermore, in line with the prediction of PKC^Pck2^-dependent regulation of Thr6 phosphorylation, PKC^Pck2^ failed to rescue the heat sensitivity of *gad8.T6A* cells (figure 6*e*). In contrast, growth of the *gad8.T6D* and *gad8.S546A* mutants following heat stress was enhanced by PKC^Pck2^ expression (figure 6*e*). Interestingly, the *gad8.T6D* and *gad8.S546A* mutants both have reduced inhibitory Tor1.T1972 (TORC2) phosphorylation (figure 4*a*,*b*). Therefore, elevated PKC^Pck2^ expression in combination with elevated TORC2 activity may be stimulating growth during heat stress.

It is well established that protein kinase C regulates actin organization in both yeast and mammals [44,45]. *PKC^pck2+^* ectopic expression led to a 30% reduction in the frequency at which cells executed NETO (re-arrangement of the F-actin cytoskeleton; figure 6*f*). Importantly, this PKC^Pck2^ expression had no impact on the ability of *gad8.T6A* cells to execute NETO (figure 6*f*). Thus, the PKC^Pck2^-dependent decline in the ability of cells to initiate the actin re-modelling that underpins growth at the new cell tip is also Gad8 Thr6-dependent.

## 3. Discussion

Gad8 Thr6 constitutes a novel PKC consensus phosphorylation site conserved in a subset of AGC kinases, including SGK1/2 in mammals and Ypk1 and Sch9 in *S. cerevisiae*. Gad8 Thr6 phosphorylation reduces Gad8–TORC2 binding. Thus, Gad8 Thr6 phosphorylation enhances TORC2 activity (figure 5*a*) because it blocks the ability of Gad8 to bind and place the inhibitory phosphate at position T1972 of Tor1 (figure 4*a*) that downregulates TORC2 activity [15]. Reduced Gad8–TORC2 binding in the *gad8.T6D* mutant also diminishes TORC2 phosphorylation of Gad8 S546 (summarized in figure 6*g*).

The impact of PKC control of Thr6 phosphorylation is highlighted by the *gad8.T6A* mutant. Because Gad8 and TORC2-Sin1 binding is increased in this mutant, at first it seems surprising that Gad8-controlled Tor1 T1972 phosphorylation and TORC2-controlled Gad8 S546 phosphorylation are not significantly increased. However, the explanation probably lies in the opposing nature of the TORC2 and Gad8 regulation. In this model, the absence of Thr6 regulation in the *gad8.T6A* mutant probably promotes a futile regulatory cycle. More specifically, reduced TORC2 activity (due to increased Tor1 T1972 phosphorylation) will reduce Gad8 activity and thereby reduce Gad8-controlled inhibition of TORC2. Consequently, the impact of either of the two phosphorylation events is abolished, to result in unchanged ‘steady-state’ activities of both kinases. Therefore, the PKC control of Thr6 phosphorylation can disrupts this futile regulatory cycle and thus activates TORC2 by blocking Gad8-mediated feedback inhibition (figure 6*g*).

The impact of Gad8 Thr6, S546 and *kinase dead* mutants are summarized in table 1. We find that Thr6 phosphorylation alters cell and vacuolar size, G1 arrest and mating as a result of the reduction in activating TORC2-controlled Gad8.S546 phosphorylation, which stems from the decline in TORC2 and Gad8 association. In contrast, Gad8 Thr6 regulation acts independently of Gad8 S546 phosphorylation in actin remodelling (NETO), cell survival following heat stress (cell integrity) and potassium stress (table 1). At present, it is unclear why some Gad8-dependent phenotypes are Thr6-dependent but Ser546 independent. As mentioned previously, phosphorylation of the T-loop of Gad8 by PDK1^Ksg1^ activates the kinase, whereas additional TORC2-dependent phosphorylation, within its carboxy-terminus, further activates kinase activity [13,14]. All of the Thr6-dependent phenotypes are sensitive to lack of Gad8 kinase activity (table 1), suggesting that at least some level of Gad8 activity is required. However, the *gad8.T6A* mutant, which is sensitive to potassium and heat stress, does not appear to have altered Gad8 or TORC2 activity. Therefore, the *gad8.T6A*-dependent phenotypes might be a result of reduced binding of Gad8 to an as yet unidentified substrate.

Finally, the Thr6-dependent phenotypes, survival following heat stress (cell integrity), potassium stress and actin remodelling, are all functions that have previously been ascribed to the human Gad8 homologues SGK1/2 [28,46,47] in which Thr6 is conserved (figure 6*a*). Importantly, mTORC2 and human PKC are also well known regulators of actin remodelling, crucial for cell migration [10,28,45]. Therefore, the Thr6-dependent regulation of the actin cytoskeleton is likely to be conserved in mammalian cells. In addition, hyperglycaemia (high blood sugar level) has been shown to activate PKC [48]. Furthermore, elevated PKC and mTORC2 activities, along with decreased SGK1 activity, have recently been associated with insulin resistance in mammalian cells [49–52]. Interestingly, the novel mode of AGC kinase and TORC2 regulation that we present here predicts that PKC-induced phosphorylation of SGK1 Thr5 would promote mTORC2 activity and reduce SGK1 activity. Therefore, PKC and elevated SGK1 Thr5 phosphorylation may contribute to insulin resistance through control of mTORC2 and SGK1 activities. Together, our data suggest that environmentally induced PKC can indirectly boost TORC2 activity by preventing AGC kinase feedback inhibition of TORC2 (figure 6*g*).

## 4. Material and methods

### 4.1. Strains

Strains used in this study are listed in the electronic supplementary material, table S1. Unless otherwise specified, cells were cultured at 28°C in Edinburgh minimal media [21] using 20 mM l-glutamic acid (EMMG) as a nitrogen source. Cells were grown exponentially for 48 h before being harvested at early exponential phase of 1.5 × 10^6^ cells ml^−1^.

To assay sexual differentiation, 4 × 10^6^ cells were mixed with equal numbers of cells of the opposite mating types and the mixture spotted onto sporulating agar before incubation at 30°C. Mating efficiency was determined at the indicated time by 2 × zygotes/[cells + (2 × zygotes)]. Nitrogen starvation was applied as follows. Cells were cultured at 28°C in minimal sporulating liquid media (MSL) [53] to a density of 1.5 × 10^6^ cells ml^−1^ and filtered into MSL minus nitrogen source.

For stress sensitivity spot test assays, cells were grown in yeast extract media (YES) to a cell density of 1.5 × 10^6^ cells ml^−1^. Ten-fold dilution series starting with 5 × 10^4^ cells were spotted on media indicated.

### 4.2. Cell length and division ratio measurement

Cells were grown at 28°C in EMMG to 1.5 × 10^6^ cells ml^−1^ and filtered into media using 20 mM l-proline as nitrogen source (EMMP). Cells were harvested at indicated time point, fixed with 3% formaldehyde, washed with PBS and stained by calcofluor. Images of cells were obtained using a CoolSNAP HQ2 CCD camera and processed with ImageJ. The ratio of cells undergoing NETO was estimated from calcofluor-stained cells (growing cell tips stain brighter with calcofluor). Cell length at division was measured (in each analysis, more than 200 cells were measured/counted).

### 4.3. Vacuolar staining

In order to analyse vacuolar morphology, cells were grown to a density of 1.8 × 10^6^ cells ml^−1^ before FM4-64 dye 5 mg ml^−1^ was added to the culture in the ratio 1 : 10 000 (dye : culture, v/v) and cells were incubated for a further 30 min before images of the living cells were captured with a CoolSNAP HQ2 CCD camera and processed with ImageJ. For each culture, at least 500 vacuoles were measured.

### 4.4. Molecular manipulations and generation of single point mutations

Standard site-directed mutagenesis was used to generate *gad8* point mutations, and the mutated *gad8* allele was introduced into cells through transformation. The recombinant gene was then used to replace the *ura4^+^* gene in the *gad8::ura4^+^.* The resulting strains were back-crossed, and prototroph progeny was selected. The presence of the mutation was verified by PCR. Thus, all *gad8* point mutations used in this study are single point mutants integrated into the *gad8* locus, and they are all prototrophic strains.

For generation of the Tor1–GST fusion substrate in the Gad8 kinase assay, Tor1 was amplified using the following primers, 5′-CTCGGATCCATCTCGCATTTCCATCACACTTTCGAAG-3′ and 5′-ACGCGTCGACAAGTCTCCAATTAATCAAAGGGTCATAG-3′, and cloned into pET-41a(+) vector. Tor1–GST was expressed in *E. coli* BL-21 cells. Pck2 was amplified by PCR from genomic wt DNA, sequenced and cloned into pRep42 plasmid to facilitate Pck2 expression from the *nmt42* promoter [41].

### 4.5. Western blotting

TCA precipitation protocol was followed for total protein extracts [54]. The following dilutions of antibodies were used in this study. 1/100 anti-Tor1, 1/100 anti-P-Tor1.T1972, 1/100 anti-P-Tor2.S1975, 1/1000 anti-P-Gad8.S546, 1/100 anti-P-Gad8.T387, 1/100 anti-Gad8 antibodies, 1/2000 phospho-(Ser/Thr) Akt substrate (PAS) antibody (Cell Signalling) and 1/1000 S6 antibody (Abcam). Anti-p-Tor1.T1972, anti-P-Gad8.S546 and anti-P-Gad8.T378 were all generated by Eurogentec. Alkaline phosphatase coupled secondary antibodies were used for all blots followed by direct detection with NBT/BCIP (VWR) substrates on PVDF membranes.

### 4.6. Large-scale Gad8 immunoprecipitation for mass spectrometry analysis

A 20 l of fission yeast culture at 3 × 10^6^ cells ml^−1^ were harvested and re-suspended in 20% TCA. Cells were disrupted using a Spex6870 freezer mill in liquid nitrogen. After washing with 0.1% TCA, the sample was re-suspended in sample buffer (80 mM Tris pH 7.5, 5 mM DTT, 5 mM EDTA) plus 2% SDS with 3 min boiling. About 4.5 volumes of sample buffer plus 1% triton X-100 were added to the supernatant. The mix was centrifuged at 10 000*g* for 5 min. IP buffer (0.5% Doc, 50 mM NaF, 0.2 mM Na_3_VO_4_, 20 mM Na-β-glycerophosphate, 1 mM PMSF and protease inhibitors) was added to the supernatant. The Gad8 kinase was immunoprecipitated on protein G dynal beads for 30 min at 4°C. The beads were then washed six times with sample buffer plus inhibitors, then heated to 80°C for 10 min prior to electrophoreses.

### 4.7. Gad8 for phospho-site mapping

Large-scale Gad8 immunoprecipitations were run on a Nupage Bis–Tris 4–12% SDS–PAGE gel (Invitrogen), The Gad8 Coomassie-stained band was excised and digested with either 20 ng sequencing-grade trypsin (Sigma-Aldrich), 400 ng LysN (Associates of Cape Cod) or 350 ng Elastase (Calbiochem) in 100 µl 40 mM ammonium bicarbonate, 9% (v/v) acetonitrile at 37°C for 18 h. The peptides were separated using a Nano-Acquity UPLC system (Waters) using a Waters NanoAcquity BEH C18 column (75 µm ID, 1.7 µm, 25 cm) with a gradient of 1–25% (v/v) of acetonitrile, 0.1% formic acid over 30 min at a flow rate of 400 nl min^−1^. The LTQ-Orbitrap XL mass spectrometer was operated in parallel data-dependent mode where the MS survey scan was performed at a nominal resolution of 60 000 (at *m*/*z* 400) resolution in the Orbitrap analyser between *m*/*z* range of 400–2000. The top six precursors were selected for CID in the LTQ at normalized collision energy of 35% using multi-stage activation at *m*/*z* 98.0, 49.0 and 32.7 Da.

### 4.8. Gad8 *in vitro* kinase assay

Tor1–GST was purified as follows. BL-21 cell expression of Tor1–GST was disrupted with lysis buffer (50 mM Tris pH 8.0, 100 mM NaCl, 10 mM EGTA, 10 mM EDTA, 0.1% Triton, 1 mM DTT, 1 mM PMSF, protease inhibitor cocktail). The supernatant was incubated with glutathione sepharose for 2 h at 4°C. The sepharose was washed 10× with lysis buffer and Tor1–GST was eluted with elution buffer (as lysis buffer but using 50 mM Tris pH 9.6 plus 6 mg ml^−1^ glutathione). The *in vitro* kinase assay was performed as previously described [14]. Briefly, Gad8 was immunoprecipitated in IP buffer (50 mM Tris pH 7.6, 150 mM KCl, 5 mM EDTA, 1 mM EDTA, 10% glycerol, 0.2% NP40, 20 mM β-glycerophosphate, 0.1 mM Na_3_VO_4_, 15 mM PNPP, 1 mM PMSF and protease inhibitors) and resuspended in kinase assay buffer (20 mM HEPES pH 7.5, 10 mM MgCl_2_, 1 mM DTT, 20 mM β-glycerophosphate, 0.1 mM Na_3_VO_4_ and 15 mM PNPP). It was mixed with 3 µg of Tor1–GST and 25 µM of ATP, incubated at 32°C for 30 min. The reaction was stopped by 5 min boiling with 2× loading buffer.

### 4.9 Tor1 *in vitro* kinase assay

*Schizosaccharomyces pombe* cells were harvested from early exponential phase cultures of 1.5 × 10^6^ cells ml^−1^. Cells were resuspended in IP buffer (50 mM Tris pH 7.6, 150 mM KCl, 5 mM EDTA, 1 mM EDTA, 10% glycerol, 0.2% NP40, 20 mM *β*-glycerophosphate, 0.1 mM Na_3_VO_4_, 15 mM PNPP, 1 mM PMSF and protease inhibitors). Following cell lysis, Tor1 was immunoprecipitated on protein G Dynal beads and used directly in *in vitro* Tor1 kinase assays. The kinase assays were performed using the K-LISA mTOR activity kit that includes substrate according to Ikai *et al.* [35].

### 4.10. Statistics

Asterisks represent statistical significance (*p* < 0.05) as determined by Student's *t*-test.

## Supplementary Material

supplmentary_info
