# Supplementary material for: Phosphorylation of the amino-terminus of the AGC kinase Gad8 prevents its interaction with TORC2
Source: Open Biol. 2016 Mar 2;6(3):150189. doi: 10.1098/rsob.150189 (PMC4821236; doi:10.1098/rsob.150189)

Supplementary Information

Figure S1. Novel phosphorylation of Gad8 at Thr6

(A) The product ion spectrum demonstrating the phosphorylation of Gad8 at T6 with annotated peptide sequence and pertinent ions labeled. The series of b ions including b2, b4,b4++, b5 and b6 (all with Mox characteristic neutral loss of 64 Da) confirm the peptide sequence. B2, b4, b4++, b5 confirm the phosphorylation is C terminal of L5 and b6 confirms the site of phosphorylation as T6 (B) Stress response of *gad8* mutants. (C) Gad8.3HA protein levels in *T6A* and *T6D* mutants. (D) Relative Gad8 levels in *T6A* and *T6D* mutants. (E-F) Gad8 protein stability in *T6A* and *T6D* mutants. Cycloheximide was added and Gad8 levels was followed over time.

Figure S2. Thr6 phosphorylation is not required for the response to nutrient stress.

(A) Nutrient stress of *T6A* and *T6D* and kinase dead Gad8 mutants to induce cell division. (B) Viability of *T6A* and *T6D* mutants were unaffected by nitrogen starvation.

Figure S3. Thr6 phosphorylation does not affect kinase activity.

(A) Gad8 *in vitro* kinase assay using Tor1 as substrate and phosphor-specific Tor1.T1972 antibodies is linear. (B) The *T6A* and *T6D* mutants did not affect Gad8 *in vitro* kinase activity.

Figure S4. Thr6 phosphorylation lies within a Protein Kinase C consensus site.

(A) Schematic of AGC kinases with the relative position of the conserved Gad8 Thr6 phosphorylation site. (B) Micrographs of heat stressed *T6A* and *T6D* mutants. (C) Heat stress response of *T6A*, *T6D* and PKC double mutants. (D) Overexpression of

Pck2 complements a *pck2.Δ* mutant. (E) Pck2 reduces Gad8.S546 phosphorylation in a *pmk1.Δ* mutant. * Indicates a background band.

Table S1. Strains used.

JP3	*h-*	lab stock
JP350	*h+*	lab stock
JP543	*h- psk1:ura4$^+$ leu1.32 ura4.d18*	(Bimbo et al. 2005)
JP598	*gad8::ura4$^+$ ura4.d18*	gad8::ura4+ from (Bimbo et al. 2005)
JP1411	*h+ gad8.S546A*	(Halova et al. 2013)
JP1545	*gad8.T6A*	This study
JP2102	*h+ gad8.T6D*	This study
JP2819	*gad8.T6AS546A*	This study
JP2820	*gad8.T6AS546D*	This study
JP2821	*gad8.T6DS546D*	This study
JP2822	*gad8.T6DS546A*	This study
JP1511	*gad8.K259R*	(Du et al. 2012)
JP2747	*gad8.T6AK259R*	This study
JP2748	*gad8.T6DK259R*	This study
JP2773	*h- gad8.T6DK259R ura4.d18*	This study
JP3125	*gad8.S546A ura4.d18*	This study
JP3126	*gad8.T6DS546A ura4.d18*	This study
JP2835	*gad8.S546D*	This study
JP2851	*h+ gad8.S546A*	This study
JP2619	*psk1::ura4+ ura4.d18*	(Bimbo et al. 2005)
JP438	*h+ pmk1:ura4+ ura4.d18*	(Bimbo et al. 2005)
JP2846	*gad8T6A sin1.13myc*	This study *sin1.13myc* from (Tatebe et al. 2010)
JP2345	*gad8T6D sin1.13myc*	This study
JP1992	*gad8T6A-3HA:kanMX*	This study
JP2172	*pck1::ura4+ ura4.d18*	(Bimbo et al. 2005)
JP2174	*pck2::ura4+ ura4.d18*	(Bimbo et al. 2005)
JP2712	*gad8.T6D pck1::ura4+ ura4.d18*	This study

JP2850	*gad8.T6D pck2::ura4+ ura4.d18*	This study
JP2623	*gad8.T6A pck1::ura4+ ura4.d18*	This study
JP2625	*gad8.T6A pck2::ura4+ ura4.d18*	This study
JP2970	*sin1.13myc ura4.d18 +*<br>*pJP642: nmt42-pck2*	This study
JP2971	*sin1.13myc ura4.d18 +*<br>*pJP5: nmt42-empty*	*sin1.13myc* from<br>(Tatebe et al. 2010)
JP2201	*gad8T6A ura4.d18*	This study
JP2202	*gad8T6D ura4.d18*	This study
JP2585	*gad8.T6A mcherry-lifeact*	*mcherry-lifeact* from<br>(Huang et al. 2012)
JP2587	*gad8.T6D mcherry-lifeact*	This study
JP2640	*mcherry-lifeact*	This study

Bimbo A, Jia Y, Poh SL, Karuturi RK, den Elzen N, Peng X, Zheng L, O'Connell M, Liu ET, Balasubramanian MK et al. 2005. Systematic deletion analysis of fission yeast protein kinases. *Eukaryotic cell* **4**: 799-813.

Du W, Hálová L, Kirkham S, Atkin J, Petersen J. 2012. TORC2 and the AGC kinase Gad8 regulate phosphorylation of the ribosomal protein S6 in fission yeast. *Biology Open* **1**: 884-888.

Halova L, Du W, Kirkham S, Smith DL, Petersen J. 2013. Phosphorylation of the TOR ATP binding domain by AGC kinase constitutes a novel mode of TOR inhibition. *The Journal of cell biology* **203**: 595-604.

Huang, J., Huang, Y., Yu, H., Subramanian, D., Padmanabhan, A., Thadani, R., Tao, Y., Tang, X., Wedlich-Soldner, R., and Balasubramanian, M.K. (2012). Nonmedially assembled F-actin cables incorporate into the actomyosin ring in fission yeast. The *Journal of cell biology 199*, 831-847.

Tatebe H, Morigasaki S, Murayama S, Zeng CT, Shiozaki K. 2010. Rab-family GTPase regulates TOR complex 2 signaling in fission yeast. *Curr Biol* **20**: 1975-1982.

Supplementary Figure 1

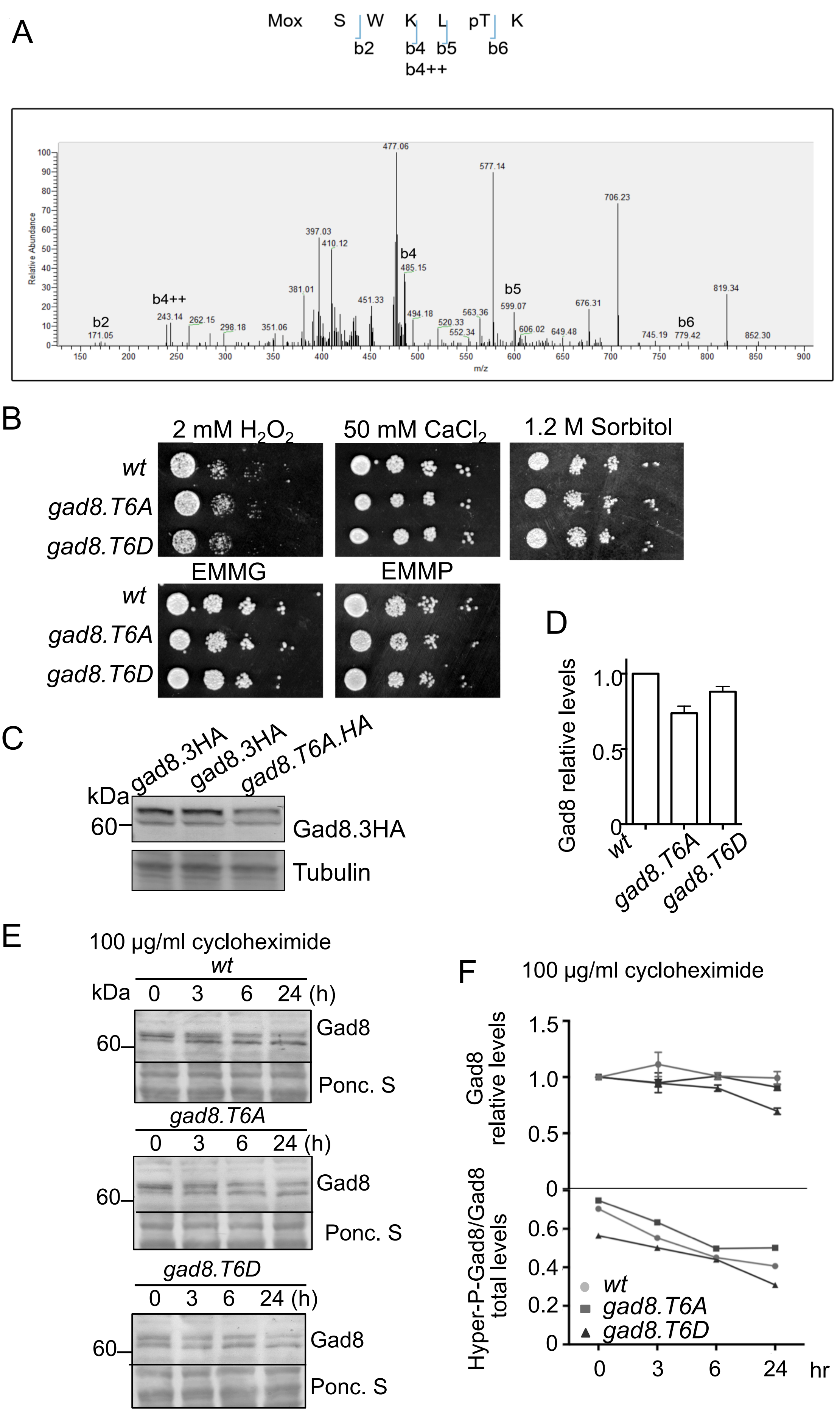

Supplementary Figure 2

A

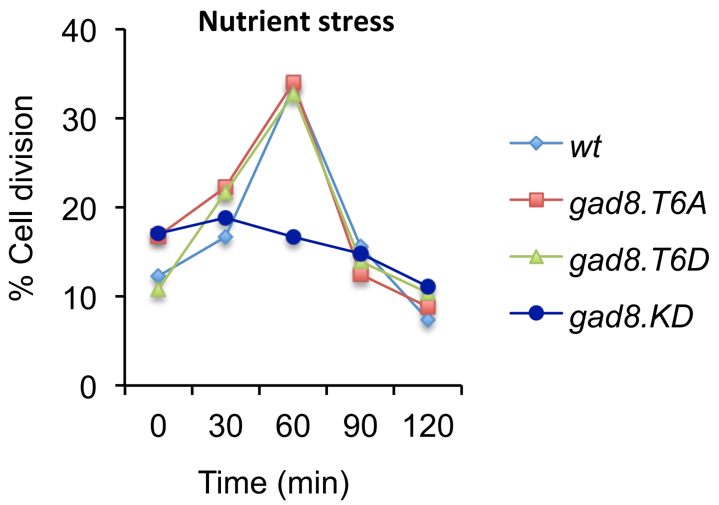

B

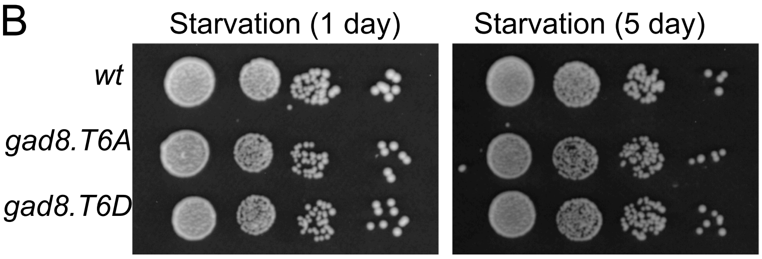

Supplementary Figure 3

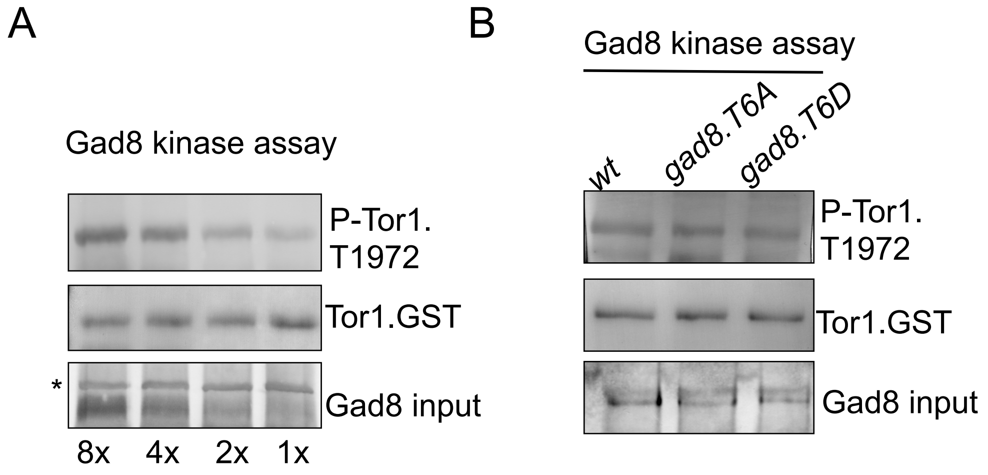

Supplementary Figure 4

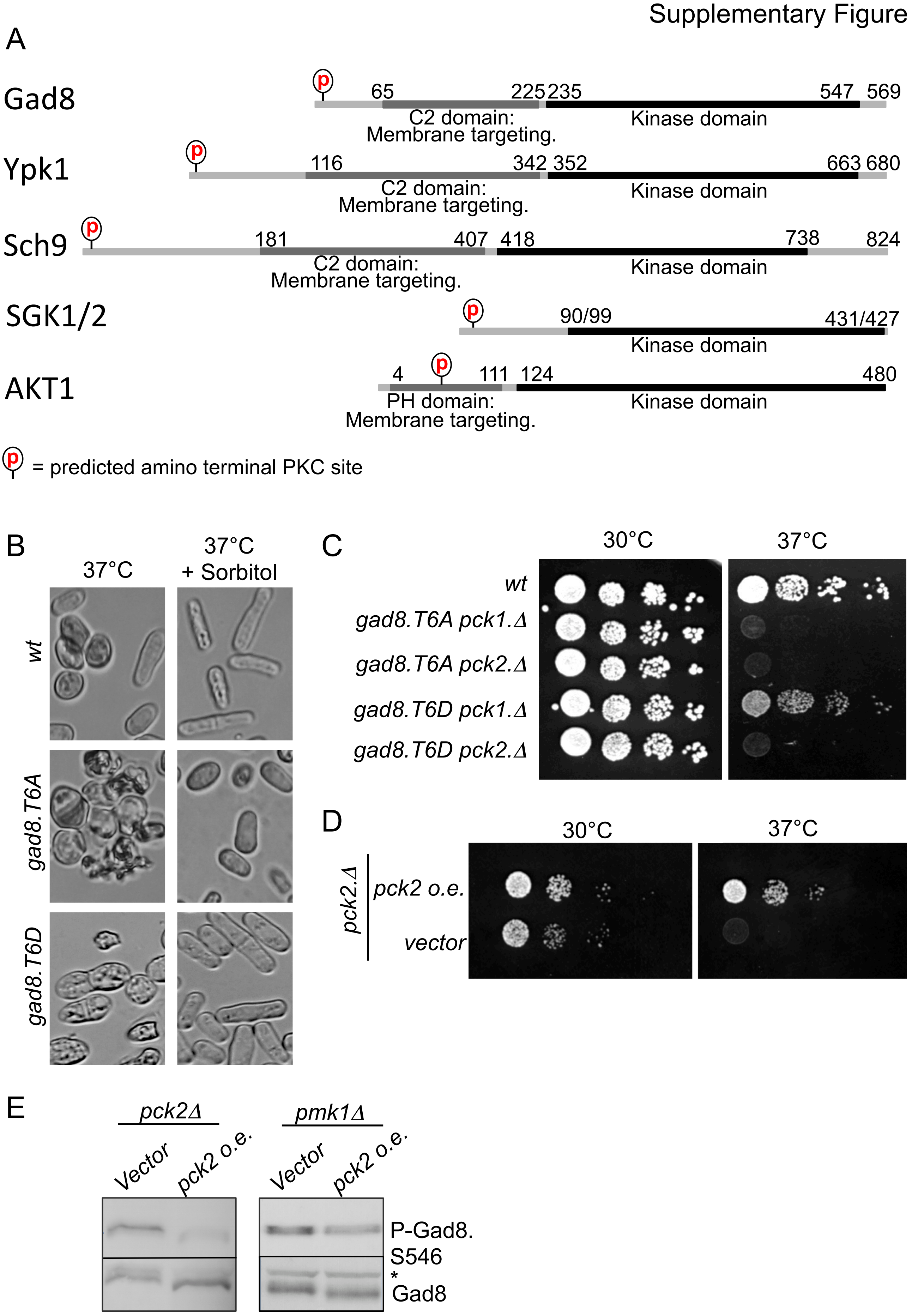

Supplement: supplmentary_info [file rsob150189supp1.pdf]
